# Folliculogenesis and acquisition of oocyte competence in cows

**DOI:** 10.21451/1984-3143-AR2019-0038

**Published:** 2019-10-23

**Authors:** Marc-André Sirard

**Affiliations:** Département des Sciences Animales, Faculté des sciences de l’agriculture et de l’alimentation, Université Laval, Québec, Canada.

**Keywords:** follicle stimulation, oocyte quality, *in vitro* maturation

## Abstract

IVF success depends on hundreds of factors and details but the oocyte quality remains the most important and problematic issue. All antral follicles contain oocytes and all of them have that have reached their full size, can be aspirated, can mature and can be fertilized in vitro. But only a few will make it to embryo unless harvested at a very specific time/status. The conditions impacting the oocyte competence are essentially dependant on the follicular status. Growing follicles contains oocytes that have not completed their preparation, as they are still writing information (RNA), later, dominant follicles or follicles at the plateau phase, stop transcription and become candidates for development. Once in transcriptional arrest, the oocytes, if not ovulated in a short amount of time, do not always make good embryos. This window is affected by time and follicle size and looks like a bell curve. The following review further explain the physiological and molecular evidences that we have to illustrate the competence window and provides clues on how to optimize ovarian stimulation to maximise oocyte quality.

## Introduction

The issue of oocyte quality in large mammals has been a major research focus since the beginning of IVF in the early eighties. The first observation of a variable quality came when oocyte obtained after ovarian stimulation using laparoscopy (Sirard and Lambert, 1985) were compared to oocyte obtained from non-stimulated cows at the slaughterhouse (Sirard and First, 1988). Already at the end of that decade, fertilization rates and culture conditions were sufficiently good that we knew that the blastocyst rate was directly dependent on oocyte origin. Although it was believed that this quality could be enhanced by in vitro culture following artificial meiotic arrest (Sirard and First, 1988), we rapidly realised that it was more complicated than just allowing more time in vitro. Thirty years later there is still an incomplete understanding of the all changes that occurs in the follicles leading to the modification of oocyte capacity to develop to the blastocyst stage but we are getting closer. One reason it took so long comes from the fact that oocytes do not contain enough material for most biochemical analytic methods and possesses a distinct physiology from most somatic cells. Protein characterization to compare oocyte with known developmental competence lead to very limited indicators of where to start the quest for a mechanism of oocyte competence (Sirard *et al.*, 2003). Even new powerful protein profiling methods require ug of proteins and a complete known proteome which is only available for somatic cells of model species or human. To add to the problem, it is quite difficult to obtain very competent oocytes to compare them to incompetent as a basis for mechanistic analysis. Indeed, the most competent oocyte available are the ones just about to be ovulated in a natural cycle of an unprimed cow and this count as 1 where we need hundreds to make any serious analysis even with the power of genomics. Surely, we can do individual cell RNAseq but the coverage will be incomplete and the RNA level of any gene in oocyte is a poor indicator of the associated function since it may be in the “stored” format. In somatic cells, the RNA as a half-life of a few minutes/hours while in oocyte an important fraction of RNAs are de-adenylated and stored (Tremblay *et al.*, 2005; Gohin *et al.*, 2014) Therefore, when a complete RNA analysis (transcriptome) is done (Robert *et al.*, 2011) the results are completely masked by the ignorance of the timing of translation of RNAs into proteins (Gilbert *et al.*, 2010) notwithstanding the fact that many proteins need a post translational modification to be active and such transformation needs hundreds of oocytes to be observed at a chemical level. In oocytes, as somatic cells, we easily observe 12000-15000 different transcripts coding for different proteins and without the capacity to distinguish their dynamics, the complexity of the analysis becomes overwhelming (Labrecque *et al.*, 2013, 2016; Labrecque and Sirard, 2014). Studies to identify the translated RNAs has been done on very specific stages (GV and MII) using large amount of slaughterhouse oocytes (Gohin *et al.*, 2014) but not on highly competent compared to less competent ones.

Unfortunately, the oocyte competence is not a single instantaneously event but a progressive transformation that occurs in a matter of days in the last part of the follicular wave (Sirard, 2016). The use of ovarian stimulation has been instrumental to learn more about oocyte quality and to obtain more material to study these cells. The initial dissection of the late folliculogenesis effect on oocyte quality was done initially by giving a few days of FSH and then slaughtering the animals to recover the ovaries to dissect the follicles and obtain oocyte from specific conditions (Blondin *et al.*, 1996; 1997a). The first big surprise was the fact that actively growing follicles from 5 to 15 mm contains oocyte of poor developmental competence when compared to slaughterhouse oocyte from ovaries obtained at random and using follicles from 2-6mm (Blondin and Sirard, 1995). This observation was key to launch the next series of investigations to explore how the time post FSH arrest is important to drive the differentiation program in the follicles and induce oocyte competence. The analysis of follicles according to their level of atresia also reveal a quite astonishing reality: healthy growing follicles (2-6mm) contains oocyte of lower quality than early atretic follicles, as measured by the blastocyst rates (Blondin *et al.*, 1997b) Not only follicle size is not in a linear relation with oocyte competence but the less actively growing follicle are better that the more actively growing at about any size. Another observation that was instrumental to build the following protocols is the fact that oocyte aspirated from ovaries obtained immediately after death contains oocyte with less competence than if a period of 4 hours is used to hold the ovaries at body temperature before harvesting the oocytes (Blondin *et al.*, 1997c). What may be happening post-mortem that improves oocyte quality? It remained a mystery for 20 years but recently we discovered that the cumulus cells would release important information in the form of specific RNAs that would be loaded into the transzonal projections during the post-mortem time (Macaulay *et al.*, 2016) and then release into the oocyte by an exosome-like system of communication (Macaulay *et al.*, 2014). As explained in a classic review paper (Sirard 2006), the oocyte competence can be divided in 3 different parts; the capacity to resume meiosis which is acquired by the early antral stage; the cytoplasmic maturation which is triggered by removing the oocyte from the follicle or by the LH surge in vivo and ; the molecular maturation which encompass the accumulation of specific information in the form of gene transcripts (RNA) for the management of the several days that the oocyte-early embryo will have to do before the activation of the embryonic genome at the eight cell stage (Vigneault *et al.*, 2004).

The understanding of the nature of cytoplasmic/molecular maturation has progressively changed the methods to control ovarian stimulation to take advantage of the effect of time (Blondin *et al.*, 2002; Sirard *et al.*, 2006). The success rate has continued to improve and we can now observe quite remarkable embryo rates following OPU, IVF and in vitro culture from cows or heifers (Landry *et al.*, 2016a; Morin-Doré *et al.*, 2017). Such improvement in the quality of embryo generated comes from a better understanding of the follicular dynamics and the basic physiological events taken place during the last few days of folliculogenesis. The following text will summarize the journey taken to improve egg quality in dairy cows.

## Results and discussion

### The concept of follicular coasting

In a natural oestrous cycle, the rise of FSH at the beginning of each follicular wave is followed by the recruitment of a dominant follicle and within a few days, the dominant follicle will increase the negative feedback through rising inhibin and estradiol levels will cause an acute decrease in circulating FSH. The dominant follicle will survive this decrease in FSH level by developing sensitive LH receptors that will maintain the growth and prevent atresia, compared to the subordinate follicles that will stop and progressively enter the atresia process within 1-2 days. This period of time during folliculogenesis seems important to prepare the granulosa cells for the major change that will occur at ovulation, the modification from an epithelial cell type into a mesenchymal cell type within the future corpus luteum (Khan *et al.*, 2016a). If the basal LH is removed by using GnRH antagonist, a different gene expression condition will emerge (Sirard, 2016) and oocyte quality will decrease rapidly as in atretic follicles (Nivet *et al.*, 2017). Other experiment testing the quality of oocyte have also confirmed the importance of follicular size to generate blastocysts post fertilization although the effect of early atresia or plateau phase seems more important than size for preparing the oocyte to develop post fertilization (Blondin and Sirard, 1995; Blondin *et al.*, 2002; Nivet *et al.*, 2012). The combination of these 2 sources of information: the different follicular condition under basal LH growth and the beneficial effect of the plateau phase in antral follicles, was instrumental in understanding the importance of reducing FSH for a define period before harvesting the oocytes. It seems that as long as FSH is driving the growth of the follicle, the oocyte does not reduce its transcriptional activity (see below) and does not begin the final preparation leading to ovulation. It is only when FSH decrease that the oocyte will either be programmed by a dominant follicle under basal LH or will start a pseudo-chromatin compaction leading to cell death (atresia) and resorption of the follicle which happens in most cases in large mammals ovulating only one follicle per cycle. We now have molecular evidence that there is a synchrony between the 3 inner follicle components, the oocyte, the cumulus and the granulosa cells during that basal LH period resulting in the activation of the 5 principal components of the differentiation: estradiol dependant genes, TGFB1, TP53, retinoic acid dependant genes and HNF4 which are instrumental to promote the changes leading to the epithelial-mesenchymal transition (Khan *et al.*, 2016b). The value of these last few days of differentiation are demonstrated by a paper where follicles were maintained in the growth phase with FSH and not allowed to go through this low FSH period (Blondin *et al.*, 1996) and showing a markedly reduced blastocyst rate. Finally, in the early years of this century, our laboratory has designed a FSH withdrawal period to improve oocyte quality after OPU-IVF (Blondin *et al.*, 2002). After several years or playing with the concept, the optimization of the low FSH period was set at 48-62 hours in adult animals and the average rate of blastocyst obtained reached 75% with some animal producing 100% blastocysts with a complete cohort of follicles (Nivet *et al.*, 2012). Since then, thousands of calves have been produced by this approach which now allows the full potential of ART to be developed in cows (Landry *et al.*, 2016b).

### The phenomenon of chromatin preparation and condensation

This second part of the puzzle comes from the group of Alberto Luciano who made the observation that oocytes at the immature stage (GV) would show different configurations for their chromatin. In very small follicles, the chromatin is diffused and associated with an active transcription as the oocyte still accumulate transcripts to support the transcriptional arrest of 7 days from pre-ovulatory follicle to the 8 cells stage in the bovine (Sirard, 2010). As the follicle continues its growth, the chromatin starts to change and becomes more compact as GV-1, in clusters in GV-2 and as a very dense structure in GV3 oocytes (Dieci *et al.*, 2016a). These changes are associated with the decrease in transcription but also involves several changes in the histones themselves (replacement with some H3.3) (Labrecque *et al.*, 2016) and their post translational modifications (Lodde *et al.*, 2017). This transformation is similar but somewhat different than the change from the non-surrounded nucleolus (NSN) pattern to the more compact form (surrounded nucleolus SN) as the mouse acquires development competence once it reaches its full size and the SN configuration while in large mammals the process is multi-step and terminates with the pre-ovulatory period where the oocytes have a GV-2 configuration and are ready to complete meiosis rapidly. The other main difference with the mouse seems to be the fact that follicle size does not predict the GV status of the oocyte. Indeed, the distribution of GV-1-2-3 is equilibrated in the different categories of follicle size and corresponds to the growing, plateau and atretic phase of follicle development (Dieci *et al.*, 2016b).

The progressive change in the chromatin configuration is not essential for meiotic resumption as GV-1 stage are fully capable of reaching the metaphase II in culture, but the developmental competence acquisition (Sirard, 2001) is not yet completed indicating that other changes must occurs in the chromatin, or in the same period as the chromatin changes. The transcriptome analysis of the oocyte according to the GV status has been done and revealed numerous histone variants changes (Labrecque *et al.*, 2016) as well as hundreds of other modifications in the RNA content associated with genes that are stored or the ones that are translated during the maturation period (Gohin *et al.*, 2014). The difficulty with RNA analysis in oocytes is that they store numerous transcripts through de-adenylation (leaving around 25 As) and the amplification systems that are used in molecular biology normally prime on the poly A tail and does not differentiate the short (stored) vs the long polyA tails (>100 As) that are rapidly translated creating a doubt if the transcript is used during the transition between GV stages or stored for maturation or post fertilization events (Gohin *et al.*, 2014). Clearly the process is dynamic and the oocyte content is modified as the ovulation get closer. Surprisingly the changes leading to atresia and ovulation are partly similar both in the gene expression profiles and in the microscopic observation of the chromatin compactness during these 2 events (Labrecque *et al.*, 2016).

### The concept of oocyte capacitation and limited lifespan

**Figure 1:**
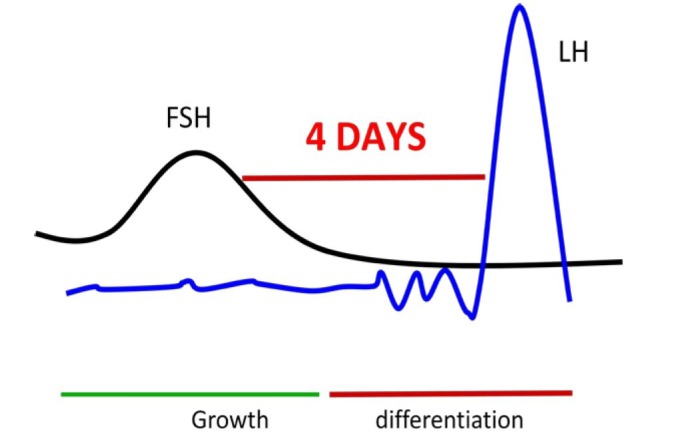
The rise and drop of FSH 4 days before ovulation

In a natural cycle, the period between the drop in FSH and ovulation varies between 4-5 days (Fig. 1). During this period, as mentioned above, basal LH maintain the growth of the follicle but also induces a different type of growth (more volume and less cell division) compared to the FSH response. The comparative analysis of gene expression from the FSH growth phase indicates that cell multiplication is reduced during the plateau (Nivet *et al.*, 2013; Douville and Sirard, 2014; Girard *et al.*, 2015) to permit progressive differentiation leading to cell secretion (ex: estradiol) and accumulation of follicular fluid to generate volume faster than tissue growth.

In bovine, the dominant follicle will regress if the progesterone remains high creating waves of follicular emergence (2 or 3 per cycle). The reason why there is a limited follicular lifespan is not completely clear as other species like human, in which the dominant normally goes to ovulation at each cycle as there is no high progesterone level to repress LH rise in response to estradiol. Nevertheless, using hormonal stimulation and antagonist blockers, the more advanced follicles in human also show a reduced oocyte quality (Nivet *et al.*, 2016). The possible explanation for the reduced lifespan is the oocyte transcriptional arrest that occurs when the chromatin compaction is completed as describe above. The ability of the oocyte to maintain homeostasis is necessarily limited with a marked reduction of the capacity to make new proteins from new RNAs. This short duration of quality may be matched by the duration of the estradiol response (high in pre-ovulatory) of the uterus which may not be sustained if no ovulation occurs. The role of estradiol in mating behavior is also important and the synchrony between follicular final growth and mating must be organized to insure the presence of sperm at or around ovulation.

### Follicles changes in natural cycles

**Figure 2:**
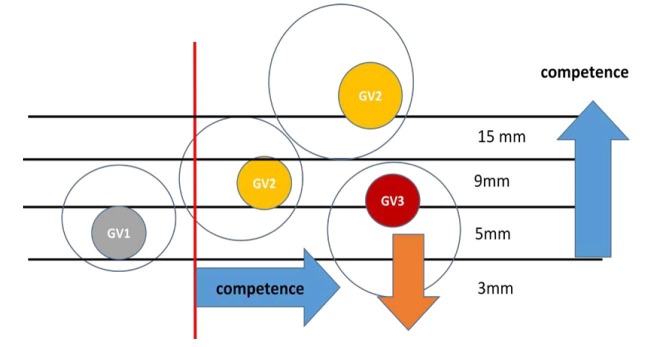
The progression of chromatin condensation in relation to follicle size and competence

To illustrate the progression of chromatin condensation as the follicles go through recruitment, selection, dominance and pre-ovulatory, the hypothetical configuration of early follicular wave is shown in figure 2. If oocytes are obtained at random at the slaughterhouse, we may expect to see a distribution where about one third of oocytes will be in the plateau phase and in the GV2 status (Dieci *et al.*, 2016b). This distribution is strikingly similar if we look at the blastocyst rate of 25 -35% for oocytes from such tissues origins.

### Follicles changes in stimulated cycles

This phenomenon of chromatin compaction and limited lifespan of the oocyte is not often considered in superstimulation treatments. When cows are stimulated for embryo flushing at day 7, FSH is used to increase the number of growing follicles and although the removal of any dominant follicle 2 days before the beginning of FSH treatment results in better synchrony of growth and the follicular distribution remains large. If we follow the same kinetics as for the natural cycle, the exogenous FSH stimulated growth will bring many follicles to the dominance status while maintaining the GV status as GV1 (Fig. 2). In such stimulated cycles, when prostaglandins are used to trigger existing corpus luteum decay and ovulation, a large range of follicular sizes remains and rarely all the oocyte are ovulated (small growing follicles do not respond to LH) and such situation results in fewer day 7 embryos than follicle counted at ovulation and even fewer embryos than CL count at day 7 casting doubts on oocyte quality in this context of follicular heterogeneity.

Although the time between FSH arrest and ovulation is shorter (2-3 days) than the natural ovulation cycle, it seems sufficient for most follicles to go through the transformation leading to a mature oocyte and the formation of a corpus luteum. Therefore, when stimulation is used with exogenous FSH prior to OPU to obtain immature oocytes without CL removal, the process of creating a plateau phase where basal LH induces the required changes in follicle and oocyte differentiation becomes important otherwise the oocytes are incompetent (Blondin *et al.*, 1996; Nivet *et al*., 2017b). In the stimulation context where all the follicles of the wave become dominant (acquire LH receptors), the mimicking of the low FSH basal LH becomes important to increase the oocyte quality. Timeline studies comparing each animal to itself while using 1-2-3-or 4 days of coasting under inhibitory level of progesterone has shown and increase followed by a decrease of oocyte quality as measured by the blastocyst rate. The optimal coasting time was set at 44-72 hours for most adult animals indicating a limited window (28 hours) of opportunity to achieve the best results (Nivet *et al.*, 2012). This period can be compared to a natural cycle when another 24 hours is added for the response to an LH surge and a little more to include the rapid pulsing of LH before the surge preparing the follicle for the final transformation. The use of this coasting protocol has increased in dairy cows where the cost procedure is justified by the embryos value and where sexing the semen makes a real economic advantage (Landry *et al.*, 2016b). Now if we continue to mimic the timeline of follicular growth and differentiation we can observed that with the coasting of all dominant follicles, the majority of them are at the GV2 stage (unpublished) at the time of collection and accordingly their blastocyst rate is close to 75%.

## Conclusion

From all these years of experimentation, it becomes clearer that the ovarian follicle is waiting for specific phases (recruitment-selection-dominance-preovulatory or atresia) to program the oocyte for transcriptional arrest and ovulation or resorption. The understanding of this process may be used to improve ovarian stimulation protocols and obtain high blastocyst rates after immature oocyte aspiration and IVP.

## References

[B001] Blondin P, Sirard MA (1995). Oocyte and follicular morphology as determining characteristics for developmental competence in bovine oocytes. Mol Reprod Dev.

[B002] Blondin P, Coenen K, Guilbault LA, Sirard MA (1996). Superovulation can reduce the developmental competence of bovine embryos. Theriogenology.

[B003] Blondin P, Guilbault LA, Sirard MA (1997). The time interval between FSH-P administration and slaughter can influence the developmental competence of beef heifer oocytes. Theriogenology.

[B004] Blondin P, Coenen K, Guilbault LA, Sirard MA (1997). In vitro production of bovine embryos: developmental competence is acquired before maturation. Theriogenology.

[B005] Blondin P, Guilbault LA, Sirard MA (1997). The time interval between FSH-P administration and slaughter can influence the developmental competence of beef heifer oocytes. Theriogenology.

[B006] Blondin P, Bousquet D, Twagiramungu H, Barnes F, Sirard M-A (2002). Manipulation of follicular development to produce developmentally competent bovine oocytes. Biol Reprod.

[B007] Dieci C, Lodde V, Labreque R, Dufort I, Tessaro I, Sirard M-A, Luciano AM (2016). Differences in cumulus cell gene expression indicate the benefit of a pre-maturation step to improve in-vitro bovine embryo production. Mol Hum Reprod.

[B008] Dieci C, Lodde V, Labreque R, Dufort I, Tessaro I, Sirard M-A, Luciano AM (2016). Differences in cumulus cell gene expression indicate the benefit of a pre-maturation step to improve in-vitro bovine embryo production. Mol Hum Reprod.

[B009] Douville G, Sirard M-A (2014). Changes in granulosa cells gene expression associated with growth, plateau and atretic phases in medium bovine follicles. J Ovarian Res.

[B010] Gilbert I, Scantland S, Sylvestre E-L, Dufort I, Sirard M-A, Robert C (2010). Providing a stable methodological basis for comparing transcript abundance of developing embryos using microarrays. Mol Hum Reprod.

[B011] Girard A, Dufort I, Douville G, Sirard M-A (2015). Global gene expression in granulosa cells of growing, plateau and atretic dominant follicles in cattle. Reprod Biol Endocrinol.

[B012] Gohin M, Fournier E, Dufort I, Sirard M-A (2014). Discovery, identification and sequence analysis of RNAs selected for very short or long poly A tail in immature bovine oocytes. Mol Hum Reprod.

[B013] Khan DR, Landry DA, Fournier É, Vigneault C, Blondin P, Sirard M-A (2016). Transcriptome meta-analysis of three follicular compartments and its correlation with ovarian follicle maturity and oocyte developmental competence in cows. Physiol Genomics.

[B014] Khan DR, Fournier É, Dufort I, Richard FJ, Singh J, Sirard M-A (2016). Meta-analysis of gene expression profiles in granulosa cells during folliculogenesis. Reproduction.

[B015] Labrecque R, Vigneault C, Blondin P, Sirard M-A (2013). Gene expression analysis of bovine oocytes with high developmental competence obtained from FSH-stimulated animals. Mol Reprod Dev.

[B016] Labrecque R, Sirard M-A (2014). The study of mammalian oocyte competence by transcriptome analysis: progress and challenges. Mol Hum Reprod.

[B017] Labrecque R, Fournier E, Sirard M-A (2016). Transcriptome analysis of bovine oocytes from distinct follicle sizes: Insights from correlation network analysis. Mol Reprod Dev.

[B018] Landry DA, Bellefleur A-M, Labrecque R, Grand F-X, Vigneault C, Blondin P, Sirard M-A (2016). Effect of cow age on the in vitro developmental competence of oocytes obtained after FSH stimulation and coasting treatments. Theriogenology.

[B019] Landry DA, Bellefleur A-M, Labrecque R, Grand F-X, Vigneault C, Blondin P, Sirard M-A (2016). Effect of cow age on the in vitro developmental competence of oocytes obtained after FSH stimulation and coasting treatments. Theriogenology.

[B020] Lodde V, Luciano AM, Franciosi F, Labrecque R, Sirard MA (2017). Accumulation of chromatin remodelling enzyme and histone transcripts in bovine oocytes. Results Probl Cell Differ.

[B021] Macaulay AD, Gilbert I, Caballero J, Barreto R, Fournier E, Tossou P, Sirard MA, Clarke HJ, Khandjian ÉW, Richard FJ, Hyttel P, Robert C (2014). The gametic synapse: RNA transfer to the bovine oocyte. Biol Reprod.

[B022] Macaulay AD, Gilbert I, Scantland S, Fournier E, Ashkar F, Bastien A, Saadi HA, Gagné D, Sirard MA, Khandjian ÉW, Richard FJ, Hyttel P, Robert C (2016). Cumulus cell transcripts transit to the bovine oocyte in preparation for maturation. Biol Reprod.

[B023] Morin-Doré L, Blondin P, Vigneault C, Grand F-X, Labrecque R, Sirard M-A (2017). Transcriptomic evaluation of bovine blastocysts obtained from peri-pubertal oocyte donors. Theriogenology.

[B024] Nivet A-L, Bunel A, Labrecque R, Belanger J, Vigneault C, Blondin P, Sirard M-A (2012). FSH withdrawal improves developmental competence of oocytes in the bovine model. Reproduction.

[B025] Nivet A-L, Vigneault C, Blondin P, Sirard M-A (2013). Changes in granulosa cells’ gene expression associated with increased oocyte competence in bovine. Reproduction.

[B026] Nivet AL, Léveillé MC, Leader A, Sirard MA (2016). Transcriptional characteristics of different sized follicles in relation to embryo transferability: potential role of hepatocyte growth factor signalling. Mol Hum Reprod.

[B027] Nivet A-L, Vigneault C, Blondin P, Sirard M-A (2017). Influence of luteinizing hormone support on granulosa cells transcriptome in cattle. Anim Sci J.

[B028] Robert C, Nieminen J, Dufort I, Gagné D, Grant JR, Cagnone G, Plourde D, Nivet AL, Fournier É, Paquet É, Blazejczyk M, Rigault P, Juge N, Sirard MA (2011). Combining resources to obtain a comprehensive survey of the bovine embryo transcriptome through deep sequencing and microarrays. Mol Reprod Dev.

[B029] Sirard MA, Lambert RD (1985). In vitro fertilization of bovine follicular oocytes obtained by laparoscopy. Biol Reprod.

[B030] Sirard MA, First NL (1988). In vitro inhibition of oocyte nuclear maturation in the bovine. Biol Reprod.

[B031] Sirard MA (2001). Resumption of meiosis: mechanism involved in meiotic progression and its relation with developmental competence. Theriogenology.

[B032] Sirard MA, Dufort I, Coenen K, Tremblay K, Massicotte L, Robert C (2003). The use of genomics and proteomics to understand oocyte and early embryo functions in farm animals. Reproduction.

[B033] Sirard M-A, Richard F, Blondin P, Robert C (2006). Contribution of the oocyte to embryo quality. Theriogenology.

[B034] Sirard MA (2010). Activation of the embryonic genome. Soc Reprod Fertil Suppl.

[B035] Sirard M-A (2016). Somatic environment and germinal differentiation in antral follicle: The effect of FSH withdrawal and basal LH on oocyte competence acquisition in cattle. Theriogenology.

[B036] Tremblay K, Vigneault C, McGraw S, Sirard M-A (2005). Expression of cyclin B1 messenger RNA isoforms and initiation of cytoplasmic polyadenylation in the bovine oocyte. Biol Reprod.

[B037] Vigneault C, McGraw S, Massicotte L, Sirard M-A (2004). Transcription factor expression patterns in bovine in vitro-derived embryos prior to maternal-zygotic transition. Biol Reprod.

